# Contrast-Enhanced Ultrasound as a Main Radiological Diagnostic Method for Primary Liver Neoplasms and Hemangiomas

**DOI:** 10.7759/cureus.18288

**Published:** 2021-09-25

**Authors:** Knkush Hakobyan, Mrunanjali Gaddam, Ugochi Ojinnaka, Zubayer Ahmed, Amudhan Kannan, Huma Quadir, Jihan A Mostafa

**Affiliations:** 1 Diagnostic Radiology, California Institute of Behavioral Neurosciences & Psychology, Fairfield, USA; 2 Internal Medicine, California Institute of Behavioral Neurosciences & Psychology, Fairfield, USA; 3 Family Medicine, California Institute of Behavioral Neurosciences & Psychology, Fairfield, USA; 4 Medicine, Jawaharlal Institute of Postgraduate Medical Education and Research, Puducherry, IND; 5 General Surgery, California Institute of Behavioral Neurosciences & Psychology, Fairfield, USA; 6 Internal Medicine/Family Medicine/Neurology, California Institute of Behavioral Neurosciences & Psychology, Fairfield, USA; 7 Psychiatry, California Institute of Behavioral Neurosciences & Psychology, Fairfield, USA

**Keywords:** infantile hepatic hemangioma, ceus, ultrasound, liver neoplasms, contrast-enhanced ultrasound

## Abstract

Contrast-enhanced ultrasound (CEUS) is a relatively new approach for the definitive diagnosis of focal liver lesions (FLL). The essential advantages of CEUS are affordability, absence of radiation, and negligible nephrotoxicity-making this diagnostic approach more preferable.

This review includes data from 39 different research studies published during the last 10 years, selected through the MeSH strategy in PubMed.

We conclude that CEUS is a promising approach for diagnosing primary liver neoplasms and it is an excellent radiological approach for children and pregnant women because of the absence of radiation and nephrotoxicity. Studies showed that CEUS is a very good approach for the differentiation of a variety of hemangiomas and for a detailed description of those findings. Therefore, CEUS is an important and progressive method for the diagnosis of liver neoplasms. The regular use of CEUS will facilitate the diagnosis of primary liver lesions.

## Introduction and background

Ultrasound is a cost-effective, real-time, widely available method for evaluating abdominal pathologies. Many medical specialists use it in their daily practice. It remains one of the best initial tests for liver and gall bladder pathologies, particularly in emergencies for liver diseases.

Usually, during a primary brightness (B)-mode ultrasound, we can find incidentalomas. Liver hemangiomas are present in 1-5% of the general population, whereas focal nodular hyperplasias (FNHs) are less popular. In contrast, focal fatty infiltrations of the liver can be seen in everyday practice [1]. Traditional ultrasound is not enough to diagnose focal liver lesions, except for cystic liver lesions, some hydatid cysts, and fatty liver lesions [2]. Contrast-enhanced ultrasound (CEUS), on the other hand, which is performed with sulfur hexafluoride microbubbles, is a real-time diagnostic method that helps make a reliable diagnosis for different liver lesions, based on contrast enhancement during arterial phase portal venous and late (sinusoidal) phase [3].

Contrast-enhanced CT and MRI have high sensitivity and specificity for focal liver lesions. However, both methods are expensive, have an extensive waiting list, and CT has radiation and nephrotoxic side effects [4,5]. CEUS is non-ionizing, has few adverse effects, and obviates the need for anesthesia, and hence is an excellent diagnostic method for children with congenital liver lesions [6]. The value of CEUS for the liver is comparable with MRI. CEUS is the only method that helps to follow neoplasm contrast enhancement in real-time during the whole examination [7].

Before performing CEUS, traditional ultrasound should be done, including both B-mode and a Doppler ultrasound. An abdominal transducer should be maintained on specific liver lesions; the particular machine should be available in a diagnostic unit to avoid microbubble disruption. The sagittal plane should be chosen to minimize motion effects and to see the lesion during the whole respiratory phase. Contrast agents are given intravenously followed by saline. The arterial phase starts approximately in 10 seconds and ends in 40 seconds after injection. The portal venous phase starts in 30 seconds and ends in 120 seconds, and later in up to five minutes starts delay or interstitial phase [8,9]. 

Hemangiomas, the most common benign finding in the general population, are easily detectable through CT or MRI. But sclerosed hemangioma is difficult to differentiate from liver cancer. However, through CEUS, with a second-generation contrast agent, Sonazoid™ (perflubutane), some differential features exist between sclerosed hemangioma and cancer as Sonazoid is phagocytized in the liver reticuloendothelial system during the sinusoidal phase [10]. Tumor cells lack reticuloendothelial (Kupffer) cells. Consequently, the malignant lesion will show a perfusion defect compared to other benign lesions [11]. CEUS is a very reliable method for differentiation between benign and malignant neoplasms of the liver [12].

This review aims to evaluate the diagnostic data that has already been collected, the value of CEUS, and summarize the most common appearance of hemangiomas by ultrasound.

The importance of this review is to show that in some cases CEUS can be as good a diagnostic method to evaluate the type of liver neoplasms as contrast-enhanced CT or MRI. 

## Review

CEUS as a diagnostic method for liver hemangiomas in children and pregnant women

CEUS is an excellent diagnostic method for pregnant women and children because no radiation is used and it is widely available. The United States (US) has approved SonoVue™/Lumason™ contrast agents for pediatric use, which is a new step for radiologic diagnoses in children [3,13]. Even though benign liver neoplasms are often seen in an adult population, these are relatively rare in children [14]. The most common types of liver diseases in children are infantile hemangioma (IH) comprising approximately 12% of all benign lesions in children [11], and congenital hemangioma (CH). Both are benign vascular tumors but pathophysiologically they have some differences. IH does not present at birth and is glucose transporter 1 (GLUT1) positive. In contrast, CH is GLUT1 negative and presents from the first day of life [6]. A study by El-Ali et al. of 10 children with either IH (five cases) or CH (five cases) has shown that in the arterial phase, both IH and CH have good contrast enhancement. In the portal phase, they remain enhanced (differential feature from metastatic deposits) and in 60% of cases, IH showed washout in the interstitial phase. Also, all the IH were hypoechoic, whereas three of five CH were heterogeneous and two of five CH were hyperechoic [6].

CEUS is also an excellent diagnostic approach for differentiating a benign hemangioma from a malignant hepatoblastoma in children [15]. Children with hepatoblastoma usually present with abdominal mass and thrombocytopenia. The enhancement of hemangiomas are centripetal, and hemangiomas are homogenous and hyper-enhanced in all phases, isoechoic in the delay phase. There is no contrast bubble destruction, which is a significant feature in ultrasound, except in the care of large hemangiomas, where central necrosis is present [11,12]. At the same time, hepatoblastomas show hyperenhancement with early washout - a typical pattern for malignancy [16].

CEUS is also helpful for post-treatment follow-up. Also, a strong correlation exists between ultrasound quantitative patterns, neoplasm size, and local invasion [17]. One study shows that a high peak in the arterial phase is associated with vascular tumors, whereas early washout has a strong association with malignancy [15].

In a study of six pregnant women by Schwarze et al., CEUS caused no adverse and harmful effects for the mother or the fetus [18].

To sum up, CEUS is a novel and maybe revolutionary method for liver neoplasms, especially in high-risk patients like children and pregnant women, because of rare side effects and the absence of radiation and nephrotoxicity. Also, CEUS can help to distinguish between malignant and benign neoplasms. In the future, CEUS will be more widely available and can be the main method for diagnosing primary liver findings.

The study in pregnant women was in a single hospital, and patient numbers were insufficient (six patients) [18]. Despite solid conviction in the advantages of CEUS in children, the sample size limits the power of the study. However, the case series study helps in some points to differentiate IH from CH and also specific malignant (hepatoblastomas) from benign hemangiomas [6].

CEUS as a diagnostic method for primary liver neoplasms

A large study comparing CT, MRI, and CEUS shows that CEUS is an excellent diagnostic method for benign neoplasm. In most cases, there is no need for correlation with other imaging methods. However, for malignant findings, sometimes correlation is needed with other imaging methods [19]. CEUS can be used as a reliable method for differentiation between focal liver lesions and malignant neoplasms [12]. Hepatic epithelioid hemangioendothelioma (HEHE) are rare, low-grade sarcomas and the diagnosis is quite tricky for radiologists [20], as well as for pathologists [21]. The study by Klinger et al. shows three main types of enhancements for HEHE: centripetal enhancement with washout, rim-like hyperenhancement with washout, and inversed target sign with washout [22]. The findings depend on the specific type of HEHE.

FNH is the second most common benign neoplasm of the liver (8-9% of all primary neoplasms) after hemangioma, more common in women [23]. The most typical findings for FNH is the central scar, which is due to the absence of vessels. FNH has a rapid, early, centrifugal arterial filling pattern, and the vessels can be seen just before nodular enhancement. FNH is also hyperechoic during portal and venous phases but becomes isoechoic in late phases. Washout is not characteristic for FNH but malignant cells demonstrate patterns of washout [24-26].

Simple hepatic cysts do not have any enhancement in any phase after CEUS. The same characteristic is found with a parasitic cyst at the beginning. As the parasitic cysts progress, calcifications can be present, and septa can be found if daughter cells exist [27]. Hepatic cysts are lesions that can be diagnosed even without contrast in regular B-mode ultrasounds.

Adenomas are benign hepatic neoplasms, primarily in women using oral contraceptives. Adenomas very rarely can become malignant. Very often, adenomas have hemorrhages. Hepatocellular adenomas (HCA) are enhanced during arterial and venous phases from the periphery (centripetal) and become isodense during late stages [28-30]. Often, adenomas show a washout pattern, and central necrosis makes a differential diagnosis from malignant carcinoma difficult [29,30]. Like FNH, HCA has a similar enhancement. Unlike FNH, however, it has centripetal enhancement. Adenomas remain challenging to differentiate from malignant neoplasm by CEUS [31]. CEUS is also sometimes helpful for the diagnosis of rare liver neoplasms [32].

So in CEUS, different focal liver lesions have some unique patterns, like washout in malignancy, centrifugal (from center to periphery) enhancement of FNH, no enhancement of cysts, and centripetal (from the periphery to center) in adenomas. In the coming times, the progression of CEUS will open more possibilities for easier and faster diagnosis of primary focal liver lesions.

A significant multicenter study was performed over a six-year period of 2062 patients who underwent CEUS, which suggested a 97.8% sensitivity, 83.9% specificity, 82.2% positive predictive value (PPV), 98.1% negative predictive value (NPV), and 89.9 % accuracy for diagnosis of benign liver lesions by CEUS (n=12) [29].

Role of CEUS in the diagnosis of hemangioma

Hemangiomas are the most common hepatic benign neoplasms among the adult population. Ultrasound without contrast in most cases is not a reliable examination for focal liver lesions with the exception of simple liver cysts. A few years ago, the only other imaging methods were CT and MRI with contrast and Technetium-99m labeled studies [2]. In the study by Sirli et al. involving 1153 patients with FLL, 238 cases of hemangioma were found using CEUS, and only 11 of them were misdiagnosed. CT/MRI or biopsy found another 24 hemangiomas. This study shows 90.4% sensitivity, 98.8% specificity, 95.4% PPV, and 97.4% NPV, resulting in 96.9% diagnostic accuracy in the aspect of diagnosis of hemangiomas.

**Figure 1:**
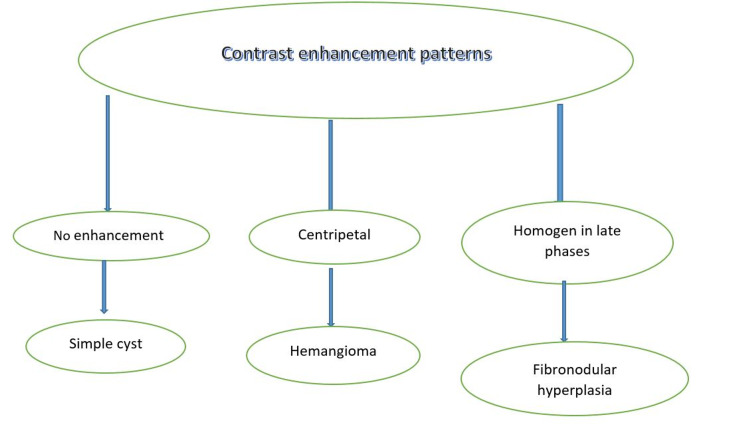
Contrast Enhancement Patterns

CEUS also has some differential diagnostic enhancement patterns for hepatic sclerosed hemangioma and malignant cells. In contrast to malignant cells, a sclerosed hemangioma can phagocytize contrast and does not show filling defects [11,33]. Typical enhancement patterns for hemangiomas are centripetal (from the periphery to the center) filling (Figure 1). This characteristic pattern widely depends on the size of the hemangioma. For example, large hemangiomas (>4cm) and giant hemangiomas (>10 cm) are rich in arteriovenous shunts and surrounded by focal hypoechoic areas, which is because of of the high amount of lipids and insulin in the liver mesenchyme, hemangiomas are supplied mainly by arteries, whereas hepatic blood comes from portal veins [34]. The specific phenomena were described in the study if the hemangioma was hyperechoic shown by an antecedent ultrasound. Unlike the usual pattern, echogenicity tends to increase after CEUS for hypoechoic lesions [35].

In a retrospective study by Quaia et al., they found no significant difference in the diagnosis of hemangioma between CEUS done with CT or CEUS alone [36]. The diagnostic value of contrast-enhanced MRI and CEUS were compared by Fang et al. for diagnosis of hemangioma: 763 total focal liver lesions were found, 80 lesions were described as hemangioma by contrast-enhanced MRI and 78 by CEUS, so there is no significant difference for diagnosis of hemangioma by contrast-enhanced MRI or CEUS [37].

Color parameter imaging (CPI) is a new analysis software for CEUS, which helps in diagnosing some types of atypical hemangiomas, especially for differentiating from liver metastatic lesions, particularly some atypical hemangiomas that have a rapid homogenous enhancement in the arterial phase. CPI helps measure the time for contrast arrival to the lesion after injection and differentiate hemodynamic features of the lesion [38].

There are studies for a specific phase with contrast agent microbubble Sonazoid, the extended phase, also called the Kupffer phase. This phase occurs long after injection since Sonazoid is phagocytized by Kupffer cells and remains longer in the liver. Study shows that hemangiomas with or without high flow rate tend to become iso- or hypotensive in the Kupffer phase [39].

Consequently, CEUS is an excellent diagnostic method even for many types of hemangiomas such as sclerosed, large, giant, and atypical hemangiomas. Also, research comparing it with CT and MRI shows almost the same diagnostic value for primary focal liver lesions, but is cheaper and has a much lower waiting time. However, the diagnostic value of CEUS is lower in patients with large body habitus and for those who have difficulties with breath-holding.

From an analytic view, the study by Sirli et al., which comprised 238 diagnoses of hemangiomas and was followed for five years, has a higher scholarly value because of the larger sample size and longer duration of the study [2], in contrast to the study by Fang et al., where only 80 patients with hemangiomas were included [37].

Limitations

This review included only research from the last ten years since CEUS is a relatively new method for diagnosing primary liver neoplasms. Moreover, some studies have a limited sample size, especially the studies among children and pregnant women.

## Conclusions

In conclusion, CEUS is a promising approach for diagnosing primary liver neoplasms and continues to actively develop as an excellent radiological approach for children and pregnant women because of the absence of radiation and nephrotoxicity. In many comparable studies with CT or MRI, there was no significant difference in making the correct diagnosis. CEUS helps distinguish benign and malignant neoplasms of the liver by contrast enhancement time and pattern differences. It also has many specific enhancement features for various types of hemangiomas. We summarized different aspects where CEUS can be used and how good diagnostic values can differentiate neoplasms.

CEUS has many advantages like being faster, cheaper, able to show Doppler blood flow in hemangioma, able to be performed at the bedside in case of unstable hemodynamics, and fewer adverse effects. Patients can get a definitive diagnosis without undergoing other radiological examinations. In the future, we would like to find more studies among the pediatric and pregnant population because these are two groups where we can see the most adverse effects with other radiologic approaches.
